# Magnetic field effects in dye-sensitized solar cells controlled by different cell architecture

**DOI:** 10.1038/srep30077

**Published:** 2016-07-21

**Authors:** M. Klein, R. Pankiewicz, M. Zalas, W. Stampor

**Affiliations:** 1 Department of Physics of Electronic Phenomena, Faculty of Applied Physics and Mathematics, Gdansk University of Technology, Narutowicza 11/12, 80-233 Gdansk, Poland; 2 Centre for Plasma and Laser Engineering, The Szewalski Institute of Fluid-Flow Machinery, Polish Academy of Sciences, Fiszera 14, 80-231 Gdansk, Poland; 3 Faculty of Chemistry, Adam Mickiewicz University in Poznan, Umultowska 89b, 61-614 Poznan, Poland

## Abstract

The charge recombination and exciton dissociation are generally recognized as the basic electronic processes limiting the efficiency of photovoltaic devices. In this work, we propose a detailed mechanism of photocurrent generation in dye-sensitized solar cells (DSSCs) examined by magnetic field effect (MFE) technique. Here we demonstrate that the magnitude of the MFE on photocurrent in DSSCs can be controlled by the radius and spin coherence time of electron-hole (e-h) pairs which are experimentally modified by the photoanode morphology (TiO_2_ nanoparticles or nanotubes) and the electronic orbital structure of various dye molecules (ruthenium N719, dinuclear ruthenium B1 and fully organic squaraine SQ2 dyes). The observed MFE is attributed to magnetic-field-induced spin-mixing of (e-h) pairs according to the Δg mechanism.

Dye-sensitized solar cells (DSSC) are devices that convert solar energy to electricity using low-cost and non-toxic materials^1^. Because of their remarkable photoconversion efficiency of over 14% reached by molecular engineering of organic sensitizers^2^ and over 21% for panchromatic dye-sensitized cell in conjunction with a perovskite cell using a system of spectral splitting^3^ this technology is becoming a credible alternative for the most popular first generation silicon-based inorganic solar cells. The transparent photoanode in the form of a mesoporous layer of a nanocrystalline wide-band gap semiconductor (mostly TiO_2_) with adsorbed monolayer of dye molecules deposited onto transparent conductive oxide (TCO) glass substrate and the counter electrode made of TCO glass coated with a thin platinum catalytic layer, between which there is an liquid electrolyte containing mostly 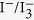 redox couple, form a typical DSSC^1^,^4^ (see Fig. 1a). The efficiency of DSSCs is limited by the electron transfer processes proceeding at the oxide semiconductor/dye/electrolyte interfaces. Among others, the charge recombination and exciton dissociation are generally recognized as the basic electronic processes limiting the efficiency of photovoltaic devices. Ultrafast electron transfer to TiO_2_ conduction band from metal-to-ligand charge transfer (MLCT) photoexcited state of Ru-bipyridyl dyes can occur from a singlet state (^1^MLCT) as well as from a triplet state (^3^MLCT) as a result of heavy metal atom induced efficient intersystem crossing (~10^−12^ s) while for pure organic dyes this electron transfer occurs efficiently only from a singlet excited state due to spin-forbidden singlet-to-triplet intersystem crossing process^5^. This primary charge separation step in DSSCs has been extensively studied by femtosecond transient absorption spectroscopy^5^,^6^,^7^, however, the exact nature of the spatial separation of charge carriers involving possibly an intermediate stage of geminate electron-hole (e-h) pairs or exiciplex states is not fully understood so far^7^,^8^. Nevertheless, if these intermediate species are endowed with the magnetic moment then a low external magnetic field of tens mT strength can interact with them and this way change the generated photocurrent as observed in tris-(8-hydroxyquinolinato) aluminum (III) (Alq_3_) films^9^, starburst amine (m-MTDATA): bathocuproine (BCP) system^10^ or in polyhexylthiophene (P3HT): [6,6]-phenyl-C61-butyric acid methyl ester (PCBM) bulk heterojunction solar cells^11^,^12^. Therefore, near-unity quantum efficiency of organic solar cells is achieved not only due to efficient (e-h) pair dissociation by electric field but it arises through the interplay between spin, energetics and delocalization of electronic excitations in organic semiconductors^13^. A recent study on electronic processes in p-type DSSC with Au nanoparticles-doped photocathode has shown that the photocurrent and photoconversion efficiency enhancement does not originate from increased absorption due to surface plasmon resonance (SPR) of electrons in nanoparticles but is induced by local electrical-magnetic field effect on electron injection process at dye-semiconductor interface^8^.

In the magnetic field effect (MFE) technique the external magnetic field causes the precession of Coulombically bound (e-h) pair spins at a frequency dependent on a field strength (B) which results in magnetic field-dependent intersystem crossing (ISC) between the singlet, ^1^(e-h), and triplet, ^3^(e-h), pair spin states. Due to different recombination and dissociation rates for these states the quantity of emissive states in the EL processes and the charge carrier population in the dissociation events in the PV devices can be changed and, in fact, they are observed as a magnetic field effect on electroluminescence (MEL), photoluminescence (MPL), conductivity (MC) or photocurrent (MPC). The MFEs occur when effective spin mixing process takes place provided that the spin coherence time of the (e-h) pairs is long enough in comparison to electron spin flip time (e.g. 2 ns in the magnetic field of 10 mT) and the electrostatic electron exchange interaction is sufficiently weak for efficient spin evolution to occur. The exchange interaction energy can be modulated by the (e-h) pair radius which is basically a distance between the electron and hole, while the pair lifetime and hence the spin coherence time can be changed by the charge carrier mobility controlled by the disorder degree of the semiconductor and defect states of the dye molecules. Nevertheless, the origin of low magnetic field effects in organic solids is currently under heavy debate. In order to clarify the MFEs previously observed in organic solar cells the following models have been proposed: (i) electron-hole pair (EHP) model^12^,^14^,^15^ involving reaction of carriers (polarons) with the opposite charge signs into excitons, (ii) bipolaron (BP) model^16^ involving reaction of polarons with the same charge signs and (iii) triplet-polaron (T-q) model^17^,^18^. In the EHP or BP models external magnetic field affects the ISC process and consequently changes singlet to triplet polaron pair (e-h or e-e, h-h, respectively) population ratio. In loosely bound polaron pairs S and T levels are quasi-degenerated, which enables efficient intersystem conversion as a result of spin magnetic dipole precession in the internal (hyperfine) magnetic field of nuclear spins. If local magnetic fields experienced by the electron and the hole are the same then the identical precession frequencies preserve the initial spin configuration. However, in the case of difference in local magnetic fields between electron and hole environment different spin precession frequencies lead to dephasing of spin magnetic dipoles. Consequently, singlet becomes a triplet, and *vice versa*. According to Zeeman effect the low external magnetic field of a few militesla competes with the hyperfine field (hyperfine interaction modulation - HFM) and thus splits the triplet sublevels, T_+1_ and T_−1_, leading to the suppression of ISC process between singlet and triplet polaron pairs^10^,^19^,^20^,^21^. However, at high magnetic field induction (typically of ca. 1 T) dephasing of spin magnetic dipoles occurs as a result of different values of Lande g factor for electron and hole entities forming (e-h) pairs which leads to the field-induced enhancement in ISC between singlet and triplet, T_0_, states - the so called Δg mechanism^20^,^22^,^23^. In the T-q model the external magnetic field competing with internal (fine) magnetic field of electronic spin origin modulates the triplet zero-field splitting (ZFS) which is usually termed as the fine structure modulation (FSM) mechanism. Based on FSM mechanism changes in carrier concentration or carrier mobility have been derived as originally proposed in Ern and Merrifield^24^ (see also ref. 21) or in trion model by Kadashchuk *et al*.^25^ (see also ref. 10 and 26), respectively. Recently magnetic field effects have been also reported for various n-types of dye-sensitized solar cells^27^,^28^, however, the proposed mechanism of these effects is unclear. The authors suggest that the observed photocurrent increase is related to EHP model which in fact should be inactive in such low magnetic fields (several tens of militesla) due to strong spin-orbit coupling induced by an orbital magnetic field of a heavy metal atom in a dye molecule^18^,^23^.

In this work we propose a detailed mechanism of photocurrent generation in DSSCs examined by MFE technique. During dissociation process of a dye sensitizer excited state, the singlet ^1^(e-h) and triplet ^3^(e-h) pairs are created where the electron occupies the conduction level of TiO_2_ and the hole is localized on an oxidized dye molecule. The external magnetic field of hundreds mT induction affects the intersystem crossing between ^1^(e-h) and ^3^(e-h) pairs and this way changes the generated photocurrent. We have observed that the magnitude of the small negative MFE on photocurrent in DSSCs is controlled by the radius and spin coherence time of (e-h) pairs which are experimentally modified by the photoanode morphology (TiO_2_ nanoparticles or nanotubes) and the electronic orbital structure of various dye molecules (ruthenium N719, dinuclear ruthenium B1 and fully organic squaraine SQ2 dyes). The observed MFE is attributed to magnetic-field-induced spin-mixing of (e-h) pairs according to the Δg mechanism.

## Results

### TiO_2_ photoanodes and dyes characterization

**Figure 1:**
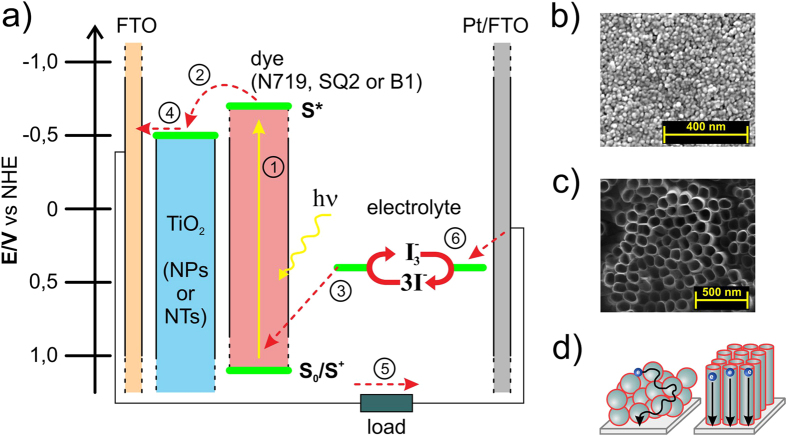
(**a**) The structure of prepared DSSCs. SEM images of (**b**) TiO_2_ NPs and (**c**) TiO_2_ NTs photoanode. (**d**) Electron diffusion path through TiO_2_ nanoparticles network (left) and ordered nanotubes (right).

**Figure 2:**
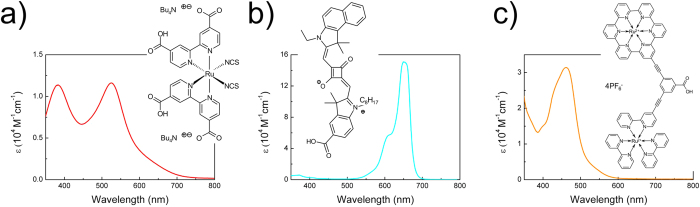
Absorption spectra and molecular structures of (**a**) N719, (**b**) SQ2, (**c**) B1 dyes in ethanol solution.

In order to carry out the experimental work a set of dye-sensitized solar cells in a typical configuration with a liquid electrolyte, TiO_2_ photoanode (in a randomly packed nanoparticles (NPs) film or highly ordered nanotubes (NTs) array form) with adsorbed dye (ruthenium N719, dinuclear ruthenium B1 or fully organic squaraine SQ2) and platinum counter electrode were prepared (Fig. 1a). TiO_2_ NPs photoanodes, with a thickness of about 11 μm were composed of nanoparticles of 8–10 nm average diameters (Fig. 1b). Prepared by two-step electrochemical anodization process of Ti metal foil, titania NTs with an average outer diameter of 90 nm and 6 μm in length were deposited onto the fluorine-doped tin oxide (FTO) glass substrates (Fig. 1c) and as such were used as TiO_2_ NTs photoanodes. Titania nanotubes offer short electron percolation pathways to charge-collecting contacts in contrast to nanoparticle matrix (Fig. 1d) while charge transport rate measured via the transient photocurrent and photovoltage decay techniques is around 10-fold slower^29^, and presumably is related to the fast trapping of free electrons occurring on time scale of a few tens of picoseconds which is at least an order of magnitude faster than in sintered nanoparticle film, and is induced by the higher concentration of shallow trap states^30^. These results suggest that different lifetimes of free electrons in TiO_2_ NT and TiO_2_ NP conduction bands should affect two factors characterizing (e-h) pairs involved as the intermediate stage of photocurrent generation process: the spin coherence time and the pair radius which is essentially the distance between a TiO_2_-trapped electron and a hole localized on a dye molecule. Namely, faster transport and longer lifetime of the free electrons in a NP layer makes the (e-h) pairs acquire greater radii. Moreover, the (e-h) pair radius will depend also on a specific orbital arrangement of the three applied sensitizers in the form of: commercially available ruthenium N719 and squaraine-based SQ2 dyes, and reported by us earlier dinuclear ruthenium polypyridine B1 dye^31^. Absorption spectra and molecular structures of these dyes are shown in Fig. 2a–c, respectively. The above mentioned ruthenium dyes exhibit the absorption bands in the visible region with maxima at 525 nm and 460 nm for N719 and B1, respectively, corresponding to MLCT transitions whereas absorption band with a maximum at 650 nm for SQ2 corresponds to 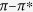 transition. We expect that both, the photoanode form and the dye structure, will be reflected in the MFE response of photocells.

### Modelling

**Figure 3:**
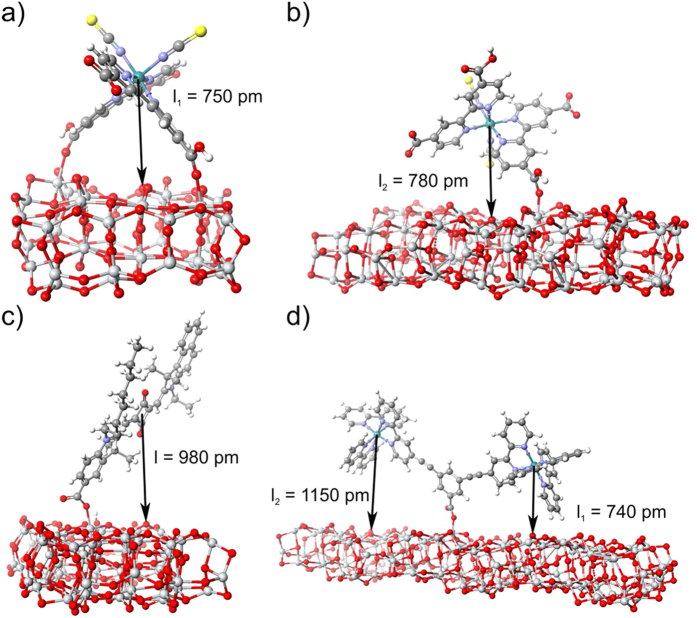
Possible binding schemes of (**a**,**b**) N719, (**c**) SQ2, (**d**) B1 to surface of anatase TiO_2_ cluster with indicated distance between the TiO_2_ surface and a central atom (**a,b,d**) or a geometrical center of hole localized on a dye molecule (**c**).

A significant role in the formation of (e-h) pairs at the interface play adsorption interaction mechanism and the geometry of the dye: semiconductor system. To find possible binding modes for all used dye molecules we have performed density functional theory (DFT) computational calculations (for details see *Methods* section). Accordingly, a distance between TiO_2_ surface and hole localized on highest occupied molecular orbital (HOMO) of an oxidized dye molecule was estimated which is in fact a minimal value of (e-h) pair radius (denoted further as l). Our results show that N719 dye may attach to TiO_2_ surface in two ways which results in formation of (e-h) pairs of different l parameters: l_1_ = 750 pm for binding simultaneously by two protonated carboxylic (opposite thiocyanate ligands) groups and l_2_ = 780 pm for anchoring by one of two deprotonated carboxylic groups (Fig. 3a,b, respectively). A dinuclear B1 dye adsorbs onto semiconductor surface anchoring by a carboxylic benzoate group but it behaves like a pan balance and due to orientational freedom (e-h) pair parameter ranges from l_1_ = 740 pm to l_2_ = 1150 pm (Fig. 3d). There is one possible geometrical orientation of SQ2 molecule binding to TiO_2_ surface which leads to formation of (e-h) pairs with a unique minimum radius of l = 980 pm (Fig. 3c).

### Magnetic field effects

**Figure 4:**
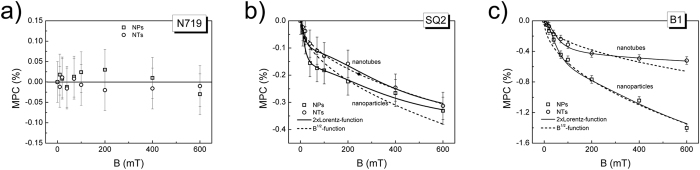
Magnetic field effect on photocurrent for DSSCs with nanoparticle/nanotube structure of TiO_2_ photoanodes sensitized by (**a**) N719, (**b**) SQ2 and (**c**) B1 dye. The MPC data points are fitted by Lorentzian function (solid lines, formula (2)) and B^1/2^-function (dashed lines, formula (3)).

To examine the influence of external magnetic field on photocurrent (the MPC signal) we have recorded the short circuit photocurrent as a function of magnetic field strength for six configurations of dye-sensitized solar cells: TiO_2_ NPs/N719 and TiO_2_ NTs/N719 (Fig. 4a), TiO_2_ NPs/SQ2 and TiO_2_ NTs/SQ2 (Fig. 4b), TiO_2_ NPs/B1 and TiO_2_ NTs/B1 (Fig. 4c). The MPC signal data points were calculated from the following formula:


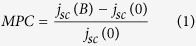


which represents a relative change of photocurrent with and without magnetic field (j_sc_(B) and j_sc_(0), respectively). For N719 dye based solar cells we have not observed any MFE within the experimental error of 0.05% whereas for B1 and SQ2 dyes a small negative MFE without saturation at the magnetic field B = 600 mT has been clearly observed. For both of them this negative MPC signal decreases when the TiO_2_ nanoparticles are replaced by the TiO_2_ nanotubes in the photoanode structure. The obtained results summarized in Table 1 indicate that the photoanode morphological architecture as well as the electronic dye structure affect magnetic field effects in dye-sensitized solar cells. In accordance with our expectations the shorter free carrier lifetime in a NT TiO_2_ conduction band translates into the shorter (e-h) pair spin coherence time and/or shorter radius, both of them reflecting in lower MPC signals for NT solar cells which suggests that there is a certain balance between these two factors. Furthermore, the predicted increase in a distance between TiO_2_ surface and a hole localized on HOMO dye molecule passing from N719 through SQ2 to B1 (Fig. 3) translates into the lower exchange interaction energy of (e-h) pairs which reveals in the more negative MFE signals (Fig. 4). For identifying the spin-mixing mechanism responsible for the observed MFEs the data points in Fig. 4 have been fitted with a double-Lorentzian function having the form of


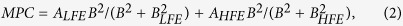


or with a single power function


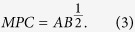


In the relevant components of the formula (2) representing the low-field (LFE) and high-field (HFE) effects A_LFE_ and A_HFE_ parameters denote the MPC signal magnitudes for 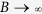, whereas B_LFE_ and B_HFE_ determine the half width (B_1/2_) at half signal maximum (HWHM)^10^. The relatively good fitting based on double-Lorentzian function (solid lines in Fig. 4) has been obtained for NP-B1 solar devices with the HWHM equal to B_LFE_ = 55 mT and B_HFE_ = 600 mT while for NP-SQ2 devices B_LFE_ = 17 mT and B_HFE_ = 400 mT. Note that the HWHM of the low field component is much broader than hyperfine field in the EHP model, which is typically ~3 mT in organic compounds^9^,^10^,^21^,^23^. Furthermore, the strong spin-orbit coupling in N719 and B1 dyes induced by the high orbital magnetic field of ruthenium atom switches off the hyperfine field-scale effects^18^,^23^, therefore, HFM spin-mixing mechanism is rather not appropriate here. Another possible alternative of observed MFE could be the T-q model in which a high triplet state concentration is of importance. Even though, for ruthenium dyes this requirement is certainly fulfilled, for a SQ2 dye after visible light absorption based on 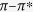 transitions prevailingly singlet excited states are formed due to inefficient ISC^32^. Moreover, in the T-q model the field-induced spin-mixing occurs at magnetic field strength comparable with the ZFS of 80 mT typical values^22^,^33^ which discards the FSM mechanism as a main origin of the observed high-field (>100 mT) effects.

To explain the MFE in analyzed solar cells the Δg mechanism could be potentially involved, wherein spin-mixing occurs as a result of different values of Lande factor for electron and hole entities constituting (e-h) pairs. Recently, this mechanism was considered for MPC effect in organic (P3HT: PCBM) photovoltaic cells (Δg is ~10^−3^)^23^ and for MPC, MPL and MEL in perovskite (CH_3_NH_3_Pb_3−x_Cl_x_) solar cell systems (Δg is ~0.65)^34^. Previous electron paramagnetic resonance (EPR) spectroscopy studies of TiO_2_^35,36^ and [Ru(bpy)_3_]^2+^ 37,38,39 have shown surprisingly different values of Lande factor for Ti^3+^ electron (g_e_) and hole [Ru(bpy)_3_]^3+^ (g_h_) radicals in comparison to free electron g value = 2.0023. It is generally recognized that Ti^3+^ is an electron center in semiconductor conduction level while on Ru(III) radical, after electron transfer from excited ruthenium dye molecule, a hole is localized. For Ti^3+^ in nanoparticles of anatase structure g = 1.988, and for a Ru(III) complex radical g = 2.63, thus for (e-h) pairs created at the photoactive solar cell interface the large value of Δg ≈ 0.64 makes the MFE be controlled by the Δg mechanism as observed recently in perovskite solar cells^34^.

The low-field and high-field components in the double-Lorentzian function could be in fact assigned to the different relaxation times of (e-h) pairs involved in dissociation/recombination processes which according to formula,


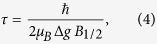


are estimated as 162 ps and 15 ps for B1, or 523 ps and 22 ps for SQ2, comparing well with those values received for (e-h) pairs in perovskite devices^34^. Nevertheless, instead of the two discrete spin-pair species a broad distribution of decay times is certainly more appropriate here in such highly-disordered nanocrystalline TiO_2_ photoanodes showing possibly non-exponential (dispersive) relaxation^23^ when an ensemble of static pairs in disordered medium is considered. However, based on spin dynamics theory of radical pair diffusion in solution^20^ the completely different scenario can be alternatively outlined where relaxation mechanism of (e-h) pairs is dominated by the diffusive motion character of charge carriers (mainly electrons in TiO_2_ film). In this case, similarly as in π-conjugated polymer: fullerene blends^40^ the high-field effects of Δg origin should be modeled by the formula (3)^20^. Indeed, the relevant fitting of MPC data in the presence of (e-h) pair diffusion (dashed lines in Fig. 4) is only of slightly poorer quality in comparison to the case of static spin pairs in non-diffusive environment.

### Mechanism

**Figure 5:**
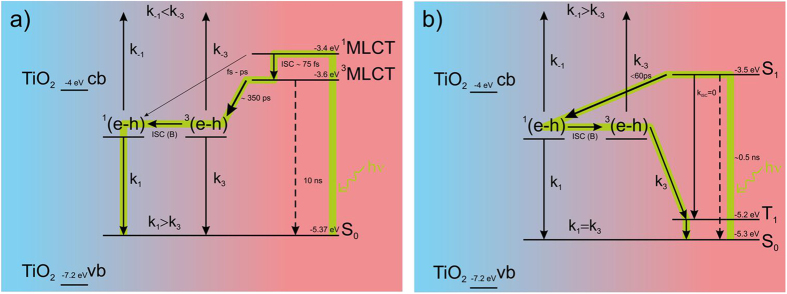
The proposed mechanism of electron transfer and charge carrier dissociation for DSSC with (**a**) ruthenium N719 or B1 dye and (**b**) organic SQ2 dye. The time constants indicated in the picture were taken from the literature^5^,^32^. The green solid lines are guide to the eyes (see online version for color images).

Regarding to our present investigation on various dye-sensitized solar cells we consider the following mechanism as a viable reason for the observed MFEs. First we describe mechanism for solar cells with ruthenium-based sensitizers depicted in Fig. 5a. In this case after absorption of solar radiation by a dye ground state (S_0_) a singlet excited state (^1^MLCT) as well as a triplet excited state (^3^MLCT), as a result of heavy metal atom induced efficient intersystem crossing (~10^−12^ s), is created. The analysis of kinetic competition between electron injection from singlet excited state (fs-ps), intersystem crossing (~75 fs), triplet state electron injection (~350 ps) and triplet state decay (~10 ns) indicates that the electron injection in N719 sensitized TiO_2_ film is dominated by injection from N719 triplet state - (^3^MLCT)^5^,^6^. Therefore, the injection processes lead mainly to formation triplet ^3^(e-h) pairs, where the electron occupies the conduction level of TiO_2_ and the hole is localized on a dye molecule. These pairs can dissociate into free carriers with k_−1_ and k_−3_ rate constants forming a photocurrent, or recombine with k_1_ and k_3_ rate constants regenerating a dye ground state, for singlet and triplet pairs, respectively. The mutual relationships between these rate constants are as follows: k_−1_ < k_−3_ due to better dissociation from triplet pairs in such a kind of heavy metal complexes^41^ whereas k_1_ > k_3_ due to more efficient spin allowed ^1^(e-h) → S_0_ recombination than spin protected transition from a triplet pair state, ^3^(e-h) → S_0_. According to the scheme (Fig. 5a) external magnetic field induces the intersystem crossing in electron-hole pairs which leads to an increase in population of singlet pairs at the expense of triplet pairs. However, the dissociation rate constant from a singlet state is less than that from a triplet state (k_−1_ < k_−3_) while the relevant recombination rate is much greater (k_1_ > k_3_), thus the generated photocurrent decreases as observed.

In the system with fully organic sensitizer the MFE mechanism depicted in Fig. 5b is slightly different. This time, due to spin forbidden molecular dye S_1_→T_1_ transition, the photoexcitation of S_1_ state is followed by an electron transfer process at picosecond time scale (<60 ps) resulting in singlet ^1^(e-h) pairs. Further, the external magnetic field induces ISC from singlet to triplet pair state forming ^3^(e-h) pairs. We should note here that in the squaraine molecule the singlet-triplet splitting energy is extremely large, ΔE_ST_ = 1.7–1.8 eV, leading to a very low position of the first triplet state, T_1_^42^. Therefore, besides dissociation, recombination pathways of a singlet pair to the dye ground state or a triplet pair to the energetically accessible T_1_ state can occur. This long lived T_1_ state relaxes to the ground state creating a crucial loss pathway which was recognized as a so-called triplet drain^43^,^44^. Thus, in this case the generated photocurrent is limited by population of singlet pairs bearing usually in organic solids higher dissociation ability^9^,^15^,^45^ in comparison to more localized triplet pairs.

To conclude, our results show that the photogeneration of free carriers in DSSCs proceeds through (e-h) pair states which play crucial role in subsequent recombination/dissociation processes. For ruthenium-based sensitizers more favorable are triplet states while for fully organic sensitizers with triplet drain this state constitutes the main source of losses. Nevertheless, in organic materials with a molecular triplet state lying higher than triplet electron-hole pair energy level dissociation from this ^3^(e-h) pair can lead to positive MFE (and in fact to an increase in generated photocurrent) as it was observed in P3HT: PCBM organic solar cells^11^ or in m-MTDATA: 3TPYMB system^43^.

## Discussion

To briefly summarize we have examined photocurrent generation processes in dye-sensitized solar cells by magnetic field effect technique. The obtained results show that charge carriers separation process occurs through the intermediate stage of electron-hole pairs for an organic dye- as well as for ruthenium dye-based solar cells. Moreover, in both cases ISC between pair spin states plays a significant role in the overall photocurrent generation mechanism. For organic dye-based solar cells triplet state dissociation is rather inefficient due to fast decay of triplets, on the contrary, the lacking triplet drain in Ru solar cells with ^3^MLCT states lying above (e-h) pairs levels ensures charge generation from triplet states to be much more efficient. This simple consideration indicates at the factors that should be taken into account when designing new sensitizers. The weak negative magnetic field effects observed in DSSCs having the various architectures are explained by the Δg mechanism ascribed to the relatively high Δg value for the electron and hole entities comprising the (e-h) pairs. Finally, we note that magnetic field effect technique is a unique tool to unravel the role of the relevant excited states and their spin mixing in charge photogeneration which is essential for any effective attempts to improve performance of new generation solar cells, in particular, dye-sensitized solar cells. With this respect the joined static magnetic field effects (MC, MEL, MPC, MPL) and reaction yield detected magnetic electron resonance (RYDMR) measurements will certainly put more emphasis on scrutiny of existing models^46^,^47^. A direct spin manipulation by a pulsed electrically detected magnetic resonance (pEDMR) has been recently demonstrated on organic materials by Boehme, Lupton and co-workers^48^. Applying combined static and pulsed magnetic field measurements on the same materials, as has been explored mainly for MEH-PPV^48^ polymer and MEH-PPV:PCBM^49^ blend offer completely new insight into existing models of magnetic effects^50^. Microscopically tracking spin polarization of interfacial organic/inorganic (e-h) pairs in DSSCs containing a liquid electrolyte and a thick semiconductor layer by pEDMR technique is really challenging.

## Methods

### Preparation of TiO_2_ NTs and NPs photoanodes

FTO substrates (7 Ω/□, Aldrich) and titanium (Ti) plates (Steam, 99.7%) were cleaned using sequentially acetone, ethanol and deionized (DI) water 10 min each in ultrasonic bath and then dried under a stream of hot air. Titania nanotubes were prepared *via* two-step electrochemical anodization of Ti plate in two-electrode configuration with platinum mesh as a cathode. The distance between electrodes was set at 2.5 cm. First anodization was conducted under 40 V for 2 h in the electrolyte containing 0.27 M NH_4_F and 1 M H_3_PO_4_ in 1/99 v/v water/ethylene glycol solution under constant temperature at 23 °C controlled by thermostat (Julabo F-12). Then, Ti plates were immersed overnight in 0.5% wt. solution of oxalic acid and then used in the next anodization. The second anodization was performed in the same conditions as first one but in the electrolyte containing 0.27 M NH_4_F in 5/95 v/v water/ethylene glycol solution. In order to remove surface debris, the titanium plate covered with nanotubes were ultrasonically cleaned in 0.05% wt. HF in DI water for 60 s. As-cleaned anodized plates were then dried at 200 °C (1 °C/min heating rate) for 1 h followed by annealing at 480 °C (1 °C/min) for 40 min. In order to detach the nanotube membrane from Ti plates, the annealed plates were anodized again in the same way but under 60 V. The obtained nanotube membranes were then transferred onto FTO substrates, pre-coated with a buffer layer, immersed in the isopropyl alcohol (IPA) filling up a Petri dish, in a similar manner to that described by Li *et al*.^51^. A 50 nm anatase buffer layer was prepared as follows: 20 μl of titanium isopropoxide (97%, Aldrich) solution in IPA with Triton X-100 (Aldrich) and acetic acid in volume ratio 1:20:4:2 were spin-coated at 3000 rpm onto FTO for 1 min and then annealed at 450 °C (10 °C/min) for 30 min. The FTO substrates covered with NT membranes were then removed from IPA and for better adhesion two drops of the above-mentioned isopropoxide in IPA solution were applied to the side of the membrane. Finally, the NTs/FTO electrodes were dried at 200 °C (1 °C/min heating rate) for 1 h followed by annealing at 450 °C (10 °C/min) for 1 h. For preparing NPs/FTO electrodes titania paste (Ti-nanoxide HT, Solaronix) was spread onto a FTO substrate using the “doctor blade” technique and sintered at 450 °C (10 °C/min) during 1 h. Both of the applied TiO_2_ electrodes consist of anatase crystalline structure, as confirmed by Raman spectroscopy.

### DSSC preparation and characterization

To prepare photoanodes titania electrodes were immersed in a 1 × 10^−4^ M solution of N719 (Solaronix) or B1 (synthesized by us^31^) dye in absolute ethanol or in a mixture of 1 × 10^−3^ M chenodeoxycholic acid (Solaronix) and 1 × 10^−4^ M SQ2 (Solaronix) dye in absolute ethanol at room temperature overnight. A platinum coated FTO was used as a counter electrode and a mixture of 0.6 M 1-butyl-3-methyl imidazolium iodide (Aldrich), 0.06 M lithium iodide (Aldrich), 0.03 M iodine (Poch), 0.1 M guanidinium isothiocyanate (Aldrich), 0.5 M 4-tert-butylpyridine (Aldrich) in acetonitrile was used as an electrolyte. The cell was assembled according to the procedure described in our previous work^31^. UV-VIS absorbance spectra of 2 × 10^−5^ M dye solutions (N719, B1, SQ2) in dry ethanol were measured by a UV-VIS spectrophotometer (Lambda 35, Perkin Elmer). The morphology of the titania electrodes was characterized by Schottky field emission scanning electron microscopy (FEI Quanta FEG 250).

### Modelling

The structures were initially optimized by semi-empirical calculations. The geometric optimization was performed by parametric method 6 (PM6) using the Scigress 2.1.0 program^52^. The DFT calculations were performed using the GAUSSIAN 03 package^53^. The geometries were optimized according to Becke’s three parameters hybrid method with the Lee, Yang and Parr exchange-correlation electron density functional (B3LYP) and 3–21G basis set.

To model the TiO_2_ nanoparticles and surfaces, we considered (TiO_2_)_38_, (TiO_2_)_76_ and (TiO_2_)_104_ clusters which were obtained by appropriate “cutting” an anatase slab exposing the (101) surface.

### Magnetic field effect measurements

For magnetic field effect measurements the samples were placed between the pole pieces of an electromagnet in a way that the magnetic field was parallel to the device plane. Magnetic field strength was controlled by an adjustable stabilized dc-power supply and a flat Hall-effect probe connected with a magnetometer (HGS-10A) placed close to the sample holder. The samples were illuminated simultaneously by two light sources: a constant white bias light from a homemade LED illuminator, composed of 14 white light emitting diodes (with a power of 30 mW each) focused onto the sample, and a single wavelength illumination setup consisted of a xenon lamp, a monochromator (Zeiss Jena) connected with a one meter-long linear quartz waveguide and an optical aperture with a diameter of 5 mm to limit the active area (photon flux of approx. 10^14^ cm^−2^s^−1^). Between a xenon lamp and a monochromator an optical chopper (MC2000, Thor Labs) was placed to modulate the monochromatic excitation light at 5 Hz frequency. Short circuit photocurrent of the solar cell was measured by lock-in amplifier (5210, EG&G Princeton Applied Research), referenced by chopper signal, connected with the sample through a current-voltage converter preamplifier (EG&G Princeton Applied Research). The monochromator output wavelength was set at 520 nm, 450 nm and 650 nm for N719, B1 and SQ2 dye based solar cells, respectively. Before magnetic measurements all solar cells were tested by a current-voltage characteristic measurement under 100 mWcm^−2^, AM 1.5 to be sure of that they work correctly and there are no internal shorts (see Fig. S1 and Tab. S1 in the Supplementary Information).

## Additional Information

**How to cite this article**: Klein, M. *et al*. Magnetic field effects in dye-sensitized solar cells controlled by different cell architecture. *Sci. Rep.* **6**, 30077; doi: 10.1038/srep30077 (2016).

## Supplementary Material

Supplementary Information

## Figures and Tables

**Table 1:** Summary of MFE results for various DSSC configurations.

Dye	HOMO hole – TiO_2_ surface distance l [pm]	MFE at 100 mT [%]		MFE at 600 mT [%]	
		NPs	NTs	NPs	NTs
N719	750/780	0.00	0.00	0.00	0.00
SQ2	980	−0.18	−0.13	−0.33	−0.30
B1	740–1150	−0.55	−0.31	−1.40	−0.50
