# Supplementary material for: Magnetic field effects in dye-sensitized solar cells controlled by different cell architecture
Source: Sci Rep. 2016 Jul 21;6:30077. doi: 10.1038/srep30077 (PMC4954973; doi:10.1038/srep30077)
Supplement: Supplementary Information [file srep30077-s1.pdf]

Supplementary Information

Magnetic field effects in dye-sensitized solar cells controlled by different cell architecture

M. Klein[1,2*], R. Pankiewicz[3], M. Zalas[3], W. Stampor[1]

[1]Department of Physics of Electronic Phenomena, Faculty of Applied Physics and Mathematics, Gdansk University of Technology, Narutowicza 11/12, 80-233 Gdansk, Poland
[2]Centre for Plasma and Laser Engineering, The Szewalski Institute of Fluid-Flow Machinery, Polish Academy of Science, Fiszera 14, 80-231 Gdansk, Poland
[3]Faculty of Chemistry, Adam Mickiewicz University in Poznan, Umultowska 89b, 61-614 Poznan, Poland
*corresponding author: mklein@mif.pg.gda.pl

Figure S1 shows J-V curves for all solar cells configurations while in Table S1 their device performance are presented. It should be noted that, the corresponding data match to the device for which MFE in Fig. 4 of the main manuscript is presented.

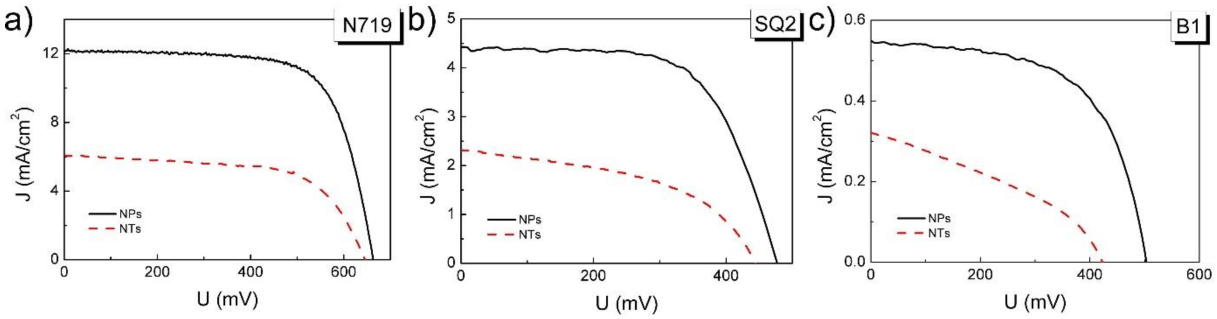

Fig. S1. Photocurrent density-photovoltage characteristic curves of the solar cells sensitized by a) N719, b) SQ2 and c) B1 dye

Table S1. Photovoltaic performance of the all dye-sensitized solar cells configurations.

Dye	J_{sc} [mA/cm2]		V_{oc} [V]		FF		PCE [%]	
	NPs	NTs	NPs	NTs	NPs	NTs	NPs	NTs
N719	12.11	6.09	0.662	0.646	0.72	0.64	5.74	2.52
SQ2	4.43	2.32	0.470	0.444	0.65	0.48	1.35	0.50
B1	0.55	0.32	0.505	0.423	0.60	0.36	0.17	0.05
